# Negative effects of accompanying psychiatric disturbances on functionality among adolescents with chronic migraine

**DOI:** 10.1186/s12883-021-02119-6

**Published:** 2021-03-03

**Authors:** Tugba Uyar Cankay, Mert Besenek

**Affiliations:** 1 Department of Neurology, Recep Tayyip Erdogan University, Faculty of Medicine, Rize, Turkey; 2 Department of Child and Adolescent Psychiatry, Recep Tayyip Erdogan University, Faculty of Medicine, Rize, Turkey

## Abstract

**Background:**

Chronic migraine is a condition with gradually increasing prevalence among adolescents which causes severe headaches resulting in functionality loss. Factors contributing to migraine becoming chronic and negatively affecting quality of life in adolescence are still unclear. Parallel with these, we aimed to examine the effect of psychiatric symptoms on headache severity and functionality loss among adolescents with chronic migraine.

**Methods:**

We evaluated features of 50 adolescents who were diagnosed with chronic migraine according to International Classification of Headache Disorders-3 for the first time in their lives by an experienced neurologist. Sociodemographic and clinical data were collected and Pediatric Migraine Disability Assessment Score, Visual Analogue Score and DSM-5 Level 1 Cross-Cutting Symptom Measure Scores (CCSM-5) were evaluated. Semi-structured psychiatric interviews were done to those who scored higher than cut-off scores on CCSM-5. Healthy control group was constituted of cases which had similar age and sex distribution to case group.

**Results:**

Majority of the case group was female (%78). There was a positive correlation between headache severity and computerized tomography history in emergency department. All of the psychiatric symptom scores were significantly higher in case group except for psychotic symptoms; but attention problems and manic symptoms clusters did not have significant difference according to the thresholds of CCSM-5. Receiving a psychiatric diagnosis did not affect frequency, severity or duration of headaches. There were also no relationship between depression/anxiety diagnosis and severity of headache/functionality loss.

**Conclusion:**

Findings suggest that; more rational treatment methods with lesser functionality loss should be developed by adopting multidisciplinary and prospective approach via psychiatric screening for adolescents with chronic migraine.

## Introduction

Headache is the most frequent somatic symptom and major cause of chronic moderately severe pains during adolescence [1–3]. Prevalence of headaches among adolescents is 3.5% and most common headache type is chronic migraine (CM) [4, 5]. CM is described as an headache with occurrence of > 15 episodes/month (≥8 episodes should have migraine features) and duration of at least 3 months according to The International Classification of Headache Disorders 3rd edition (ICHD-3) [6] . Around 69% of adolescents who refer to headache centers are diagnosed with CM [7]. Migraine rates are similar between sexes before adolescence; but after puberty, incidence rates increase for both sexes and females have higher CM prevalence during adolescence [5, 8].

Chronic pains have negative effects on cognitive functions (attention, perception, catastrophication and executive functions) as well as “state” emotional (anger, state anxiety) and “trait” emotional (clinical manifestations include depression and anxiety disorders) features [9]. Headaches causes disturbances in school attendance, healthy social interactions and youth activities by resulting in functionality loss during adolescence and can still be the major factor for impairments in quality of life during adulthood [10–13]. Recurrent headaches seen in adolescents cause both social and economical burden by increasing the frequency of referrals to neurology/psychiatry out-patient units and emergency departments [14].

Biological, cognitive, emotional and behavioral factors are known to play major roles in occurrence of headaches (especially CM) during adolescence; thus evaluation of comorbid psychiatric conditions are suggested in order to provide more comprehensive and effective treatment [15, 16]. In this aspect, previous studies have evaluated co-occurrence of psychiatric problems causing emotional dysregulation (such as aggression and irritability) and CM and demonstrated significant relationships in adolescent age group [4, 17, 18]. However, studies which extensively use DSM-5 diagnostic criteria are limited and there is no previous study in the literature which used semi-structured psychiatric interviews based on DSM-5. In line with this, we primarily aimed to compare psychiatric diagnoses/symptoms of adolescents with CM to healthy controls based on DSM-5. Our secondary aim was to evaluate the relationship between socio-demographical/ clinical/ psychosomatic features and severity of headache/ functionality loss.

## Methods

### Study design

This cross-sectionally designed case-control study included a total of 50 participants, aged between 12 and 18 with who had no previous neurological disease history and were diagnosed with CM according to Headache Questionnaire (created by neurologist based uopon ICHD-3) by an experienced neurologist [6]. Patients with pure CM were included in the study and individuals who had comorbidity of any other types of headache (e.g. tension-type headache) were excluded. The G-Power analysis program [19] was used to calculate the sample size. Type I Error 0.05, Type II Error: 0.10, 1-β (power): 90% of the sample size was calculated as 47 for each group. This research was simultaneously carried out in Neurology and Child and Adolescent Psychiatry Departments in Recep Tayyip Erdoğan University Training and Research Hospital between dates January 2019 – January 2020. Detailed physical and neurological examinations of the patients were done in headache out-patient unit; their body-mass indexes were calculated [weight (kg)/height^2^(m^2^)] and classified as normal or over-weight/obese according to Turkish standart percentiles [20]. Self and family histories of the patients were examined as well as their individual and environmental risk factors which might cause headache. Clinical backgrounds were investigated through a country-wide computerized automation system (E-Nabız) which provides access to all previous clinical examinations, blood tests and imaging modalities. Emergency referral histories, skull and sinus x-rays and computerized tomographies (CTs) were recorded. Comorbid psychiatric disorders were screened using DSM-5 Level 1 Cross-Cutting Symptom Measure Scores (CCSM-5) and individuals who scored higher than threshold scores for each symptom cluster were further evaluated by semi-structured psychiatric interviews (Schedule for Affective Disorders and Schizophrenia for School-Age Children-Present and Lifetime Version, DSM-5 [K-SADS-PL-DSM-5]) in order to determine their psychiatric diagnoses.

In order to compare psychiatric features; healthy control group (*n* = 50) was constituted of classmates of patients who were matched by gender, age and socio-economical level and did not have any headache or other psychoneurological diagnosis. Inclusion criteria were defined as, having a first diagnosis of CM (with or without aura) and no history of antidepressant or antipsychotic drug use in the last 6 months. Patients who had any other neurological disease, any other type of headache and mental retardation were excluded from the study. Informed consent was obtained from both the adolescent and her parent/legal guardian prior to the study. The study was conducted in accordance with the ethical guidelines, including the World Medical Association Declaration of Helsinki 2008, and the legal requirements of the Ethics Committee of the Recep Tayyip Erdogan Medical Faculty it was conducted in (approval no: 2020/99).

### Materials

- Socio-demographical Questionnaire: This questionnaire was created by the researches and it examines the age, gender, living place (with family or in dormitory), education level, school performance, number of siblings, socio-economical level, heating of the household, history of head trauma, smoking, dietary habits and sleeping habits.
- Headache Questionnaire: This questionnaire was created by the neurologists according to ICHD-3 criteria and it evaluates frequency, duration, characteristics, episode forms, time between start and clinical referral, co-existing symptoms, triggering factors, age of onset and familial history of headaches.
- Visual Analog Scale (VAS): This is a questioinnaire which is used to define and follow-up pain level during headache episodes. Patients score their perceived pain level between 1 (no pain) and 10 (most severe). It has been found as a suitable instrument for evaluation of adolescent CM in first diagnosis and treatment follow-ups [21, 22].
- The Pediatric Migraine Disability Assessment Scale (PedMIDAS): This is the only scale which examines migraine-related functionality loss among school-age children. It includes total of 6 items which look into number of school days missed, decreased functionality during lessons and disturbances in functionality in home or social/recreational activities. Scoring range from 1 (no functionality loss) to 4 (severe functionality loss). It is an appropriate scale to evaluate burn-out due to migraine and treatment response among adolescents [22, 23].
- DSM-5 Level 1 Cross-Cutting Symptom Measure (CCSM-5): CCSM-5 is a self-report scale which was developed to screen psychiatric symptom clusters which are important for psychiatric diagnoses [24]. Child version of CCSM-5 (which can be used between ages 11 and 18) evaluates total of 12 clusters which include; depression, aggression, irritability, manic symptoms, anxiety, somatic symptoms, attention deficit, suicidality, psychotic symptoms, repetitive thoughts and behaviors and substance abuse/misuse. Each item questions the level (or the frequency) of disturbance due to a particular psychiatric symptom in the last 2 weeks time period. Turkish reliability and validity study of CCSM-5 was done by Yalin Sapmaz et al. in 2016 [25].
- Turkish Version of Schedule for Affective Disorders and Schizophrenia for School-Age Children-Present and Lifetime Version, DSM-5 [K-SADS-PL-DSM-5]: This is a semi-structured psychiatric interview which was developed by Kaufman et al. in order to diagnose psychiatric disorders amon children and adolescent and was updated according to DSM-5 diagnostic criteria in 2016 [26, 27]. Unal et al. have done the reliability and validity study of updated version in Turkish language [28].

### Statistical analysis

Data was analysed using the Social Sciences software version 21.0 (SPSS Inc. Chicago, IL, USA). The Kolmogorov-Smirnov test was used to check whether the data were normally distributed. In order to compare data between groups; Independent T-Test and ANOVA were used for parametric data and Kruskal-Wallis (KW) and Mann-Whitney U (MWU) tests were used for non-parametric data. Categorical data were analysed with Chi-Square test. Mean [standart deviation (±SD)] values were given for parametric data, median [inter-quartile range (IQR)] values were given for non-parametric data and frequencies (percentage) were given for categorical data. Correlations between continuous data were analysed with Pearson Correlation test; where as relationship between categorical data was analysed with Spearmen Correlatino test. The value of *p* < 0.05 was accepted as statistically significant.

## Results

### Demografic features and headache characteristics

Majority of the patients were female (%78) and had mean age of 15.5 (±1.45) years. Time between emergence of headache symptoms and clinical referral was 18.8 (±21.3) months and they had mean head ache duration of 20.7 (±6.78) days per month. Median value of VAS was 7 (2) and median value of PEDMIDAS was 2 (3). Analgesic drug over-use was present among 14% of the patients and ages of analgesic over-users ranged between 16 and 18 years. Over-weight/obese patients constituted only %26 of case group. Rate of at least one other chronic illness among adolescents with CM was 16% (allergy [50%], allergy and asthma [25%], asthma [12.5%]). Majority of the referrals were during autumn (16%) and winter (20%). During the emergency department referrals; 46% of patients under-went sinus x-ray and 18% of patients under-wnt CT (Table 1). Positive correlation was found between VAS scores and history of CT during emergency department referrals (*p* = 0.011, Table 2). Anamnesis revealed that 74% of the patients had positive family history for headaches (Tablo 1). There were no significant correlations between sosciodemographical features (age, gender, parental education level, socio-economical level and family history of headache) and VAS/PEDMIDAS (Table 3).

**Table 1:** Clinical and psychiatric features of adolescents with chronic migraine

		Chronic Migraine Group (***N*** = 50)	
		Number	Percentage (%)
**Gender**	**Male**	11	22
	**Female**	39	78
**Referral Season**	**Winter**	20	40
	**Spring**	6	12
	**Summer**	8	16
	**Autumn**	16	32
**Relationship Between Referral and School**	**During school term**	40	80
	**Not during school term**	10	20
**Headache Frequency**	**< 25 days/month**	32	64
	**≥ 25 days/month**	18	36
**Genetic Load for Headache**	**In mother OR father**	30	60
	**In mother AND father**	7	14
	**None**	13	26
**Obesity**	**Over-weight/ Obese (BMI ≥ 25)**	13	26
	**Normal (BMI < 25)**	37	74
**Chronic Physical Illness**	**At least one**	8	16
	**None**	42	84
**Socio-economic Level**	**Low**	23	46
	**Moderate/ High**	27	54
**Sleep Pattern**	**Regular**	28	56
	**Irregular**	22	44
**Heating of the Household**	**Central heating (Radiator)**	30	60
	**Stove**	20	40
**History of Traumatic Head Injury**	**Present**	4	8
	**Not present**	46	92
**Dietary Habits**	**Healthy/ Regular**	27	54
	**Irregular**	23	46
**History of Psychiatric Referral**	**At least one**	14	28
	**None**	36	72
**Psychiatric Diagnosis During the Study**	**At least one**	29	58
	**None**	21	42
**Medications**	**Atidepressants (Sertraline 25–100 mg/day or Fluoxetine 10–20 mg/day)**	25	50
	**Propranolol (80 mg/day)**	10	20
	**Flunirazin (5 mg/day)**	5	10
	**Vitamin replacement**	10	20
**History of Cranial CT During Emergency Department Referral**	**At least one**	9	18
	**None**	41	82
**History of SXR During Emergency Department Referral**	**At least one**	23	46
	**None**	27	54

*BMI* Body-mass Index, *CT* Computerized Tomography, *SXR* Sinus X-ray

**Table 2:** Relationship between clinical features and Visual Analogue Scale (VAS)/ Pediatric Migraine Disability Assesment Scale (PEDMIDAS)

	VAS			PEDMIDAS		
	r	ρ	p	r	ρ	p
**Gender**		0.143	0.323		0.213	0.138
**Referral Season**		−0.147	0.308		0.037	0.798
**Referral During School Term**		−0.102	0.481		−0.004	0.980
**Genetic Load for Headache**		−0.197	0.243		−0.168	0.321
**Headache Frequency (< 25 or ≥ 25 day/month)**		0.079	0.586		0.087	0.547
**Obesity**		−0.238	0.096		−0.151	0.294
**History of Chronic Physical Illness**		0.121	0,401		−0.197	0.171
**Socio-economic Level**		0.195	0.176		−0.064	0.660
**Sleep Pattern**		0.154	0.285		0.012	0.936
**Heating of the Household**		0.040	0.782		0.100	0.489
**History of Traumatic Head Injury**		0.038	0.795		−0.106	0.462
**Dietary Habits**		−0.035	0.809		0.043	0.765
**Height**	−0.033		0.819	−0.240		0.093
**Weight**	−0.081		0.574	−0.190		0.186
**Body-mass Index**	−0.065		0.653	−0.101		0.484
**Headache Frequency (days/month)**	0.019		0.895	0.148		0.304
**Total Screen Time**	−0.294		0.038	−0.172		0.233
**Vitamin B12 Levels**	0.079		0.588	0.083		0.565
**Number of Administered Brain CTs**	0.357		**0.011**	0.192		0.182

*r* Pearson Correlation Coefficient, *ρ* Spearman Correlation Coefficient, *CT* computerized tomography

**Table 3:** Comparison of Visual Analogue Scale and Pediatric Migraine Disability Assessment Scale scores between clinical groups

		Visual Analogue Scale				Pediatric Migraine Disability Assessment Scale			
		Mean Rank	Z	df	p	Mean Rank	Z	df	p
**Gender**	**Male**	21,82	−0,998		0,318 ^a^	19,95	− 1488		0,137 ^a^
	**Female**	26,54				27,06			
**Obesity**	**Normal (BMI < 25)**	27,43	-,1664		0,096 ^a^	26,74	− 1060		0,289 ^a^
	**Over-weight/ Obese (BMI ≥ 25)**	20,00				21,96			
**Headache Fequency**	**< 25 days/month**	24,69	−0,553		0,580 ^a^	24,59	−0,610		0,542 ^a^
	**≥25 days/month**	26,94				27,11			
**Referral Season**	**Spring**	24,83		3	0,173 ^b^	27,00		3	0,919 ^b^
	**Summer**	24,63				24,75			
	**Autumn**	31,53				23,75			
	**Winter**	21,23				26,75			
**Genetic Load for Headache**	**Mother OR Father**	19,97	− 1181		0,276 ^a^	19,83	− 1006		0,350 ^a^
	**Mother AND Father**	14,86				15,43			
	**None**	26,70				26,41			
**Sleep Pattern**	**Regular**	23,63	− 1080		0,280 ^a^	25,36	−0,081		0,935 ^a^
	**Düzensiz**	27,89				25,68			
**Heating of the Household**	**Central heating (Radiator)**	25,05	−0,281		0,779 ^a^	24,37	−0,701		0,483 ^a^
	**Stove**	26,18				27,20			
**Dietary Habits**	**Regular (Healthy)**	25,94	−0,246		0,806 ^a^	24,94	−0,304		0,761 ^a^
	**Irregular**	24,98				26,15			

*BMI* Body-mass Index

^a^ Mann-Whitney-U test

^b^Kruskal-Wallis test

### Current comorbid psychiatric disorders and overall functioning

Total of 14 CM patients (28%) had a history of at least one previous psychiatric referral, total of 29 CM patients (58%) received a new psychiatric diagnosis according to K-SADS-PL-DSM-5 during the time period of the study and the most common (22%) psychiatric diagnosis was the comorbidity of Major Depressive Disorder (MDD) and Generalised Anxiety Disorder (GAD). Among adolescents with CM; total of 8 patients MDD (16%) and 6 patients received GAD (12%) diagnosis. Other Psychiatric diagnoses included; bipolar disorder (2%), obsessive-compulsive disorder (OCD) (2%), OCD and MDD comorbidity (2%) and attention deficit and hyperactivity disorder (ADHD) (2%). All of the psychiatric symptom scores in CCSM-5 were significantly higher in case group except for psychotic symptoms; but attention problems and manic symptoms clusters did not have significant difference according to the thresholds of CCSM-5 (Table 4). There were positive correlations between attention problem scores on CCSM-5 and VAS (*p* = 0.005, Pearson Correlation test) and PEDMIDAS (*p* = 0.013, Pearson Correlation test) scores. In addition there was a positive correlation between somtatic symptom scores on CCSM-5 and PEDMIDAS (*p* = 0.004, Pearson Correlation test) scores (Tablo 5). Patients who received MDD and/or GAD diagnosis did not differ from patient who did not receive these diagnoses regarding VAS (the mean ranks of psychopathology and non-psychopathology groups were 16.2 and 16.76, respectively; Z = − 0.186, *p* = 0.852) and PEDMIDAS (the mean ranks of psychopathology and non-psychopathology groups were 14.33 and 18.41, respectively; Z = − 1.291, *p* = 0.197).

**Table 4:** Comparison of DSM-5 Level 1 Cross-Cutting Symptom Measure (CCSM-5) scores between case and control groups

CCSM-5 Symptom Cluster		Case (***N*** = 50)		Control (***N*** = 50)		p^a^	Z^b^	p^b^
		Number (%)	Mean Rank	Number (%)	Mean Rank			
**Somatic Symptoms**	**Score**		69.29		31.71		**−6.557**	**< 0.001**
	**Sub-treshold**	49 (98%)		14 (28%)		**< 0.001**		
	**Above Treshold**	1 (2%)		36 (72%)				
**Sleep Problems**	**Score**		56.09		44.91		**−2.014**	**0.044**
	**Sub-treshold**	25 (50%)		8 (16%)		**< 0.001**		
	**Above Treshold**	25 (50%)		42 (84%)				
**Attention Problems**	**Score**		62.78		38.22		**−4.330**	**< 0.001**
	**Sub-treshold**	43 (86%)		38 (76%)		0.202		
	**Above Treshold**	7 (14%)		12 (24%)				
**Depressive** **Symptoms**	**Score**		65.49		35.51		**−5.223**	**< 0.001**
	**Sub-treshold**	42 (84%)		18 (36%)		**< 0.001**		
	**Above Treshold**	8 (16%)		32 (64%)				
**Irritability**	**Score**		63.50		37.50		**−4.597**	**< 0.001**
	**Sub-treshold**	39 (78%)		23 (46%)		**0.001**		
	**Above Treshold**	11 (22%)		27 (54%)				
**Agression**	**Score**		64.83		36.17		**−5.081**	**< 0.001**
	**Sub-treshold**	41 (82%)		23 (46%)		**< 0.001**		
	**Above Treshold**	9 (18%)		27 (54%)				
**Manic Symptoms**	**Score**		57.17		43.83		**−2.385**	**0.017**
	**Sub-treshold**	17 (34%)		11 (22%)		0.181		
	**Above Treshold**	33 (66%)		39 (78%)				
**Anxiety Symptoms**	**Score**		59.49		41.51		**−3.125**	**0.002**
	**Sub-treshold**	37 (74%)		26 (52%)		**0.023**		
	**Above Treshold**	13 (26%)		24 (48%)				
**Psychotic Symptoms**	**Score**		52.58		48.42		−1.073	0.283
	**Sub-treshold**	10 (20%)		7 (14%)		0.424		
	**Above Treshold**	40 (80%)		43 (86%)				
**Repetitive Thoughts/ Behaviors**	**Score**		57.91		43.09		**−2.581**	**0.010**
	**Sub-treshold**	30 (60%)		15 (30%)		**0.003**		
	**Above Treshold**	20 (40%)		35 (70%)				
**Substance Abuse**	**Present**	3 (6%)		9 (18%)		0.065		
	**Not present**	47 (94%)		41 (82%)				
**Suicidality**	**Present**	5 (10%)		3 (6%)		0.715		
	**Not present**	45 (90%)		47 (94%)				

^a^ Chi-Square test, statistically significant values are written in bold

^b^ Mann-Whitney U Testi, statistically significant values are written in bold

## Discussion

This research examined the socio-demographical/ clinical caracteristics and relationship between evironmental risk factors/ psychopathological features and headache severity/ functionality loss among adolescents with CM diagnosis. In line with previous studies, majority of the patients who were referred to hospital during the study period and received CM diagnosis were female [5]. Regarding referral season; even though previous studies in this aspect only examined acute migraine, similar to those we found majority of the referrals were done during autum and winter [29]. We failed to demonstrate a relationship between headache frequency/ severity and school performance or parental education level; which is also parallel with a previous research done by Torres-Ferrus et al. [30].

Correlation between VAS scores and history of CT during emergency department referral is a curious finding which, to our knowledge, was not reported before and it may be due to efforts of emergency physicians trying to exclude secondary headache etiologies among patients with more severe headaches. Contrary to previous research; non of the headache patients (individuals with more severe headaches included) did not stop attending their educations which may reflect some social and cultural differences can play important roles on the outcomes of CM [14, 31]. PEDMIDAS scores of our patients were relatively low (median value was 2 out of total score of 24) and we think that this might be due to differen scoring methods in previous studies and lack of Turkish language validation studies [4]. We could not find a possible correlation parental history of headache and VAS/ PEDMIDAS scores and this may be the consequence of role-model potention of parents with headaches – children might learn how to better cope with headaches from their parents [32].

Somatic, anxious, aggressive, attention deficit, irritable and depressive symptoms in CCSM-5 were significantly higer in adolescents with CM compared to healthy controls. CCSM-5 has questions about headache, nausea and vomiting in the section of somatic symptomatology and this might explain the higher somatic symptom scores seen on case group. In addition, pain is known to be a complex and multi-dimensional phenomenon which constitutes of sensorial, emotional/ motivational and cognitive components [33]. Psitive correlation between VAS and somatic symptom scores might be due to the negative effect of pain onsensorial and cognitive factors. Similarly, positive correlation between PEDMIDAS and somatic symptom socres might reflect disturbances in sensorial-cognitive mechanisms and development of over-reactivity towards pain.

Involvement of psychiatric point of view in headache field extends a long history and as a consequence, comprehensive investigation and wholesome definition of this complex phenomenon have accelerated [34]. Relationship between psychiatric disturbance and headaches include both causative and mutual natures including common genetic and/or environmental risk factors [35]. Studies have shown that, interaction between migraine and psychiatric disorders are bidirectional; one enhances the occurrence rate of other [36, 37]. Furthermore, clinicians who work in this field came to conclusion that “migraine is a special type of headache for psychiatrists” after examining its relationship to personality features and psychiatric comorbidities [38]. There are numerous studies which have examined psychiatric problems/ diagnoses and demonstrated relationship between internalization disorders (depression and anxiety) among adolescents with migraine [39, 40]. In a research done by Oztop et al. (2016), 40% of the children and adolescentswith migraine also received at least one psychiatric disorder diagnosis according to DSM-IV and these patients had significantly higher depression scores compared to healthy controls [41]. Another recent study which also used DSM-IV criteria found that, psychiatric diagnosis rates among adolescents with migraine was 56% [17]. In line with the literature, we also found that 58% of the adolescent with CM received at least one psychiatric diagnosis according to DSM-5 criteria and the most frequent diagnosis was comorbidity of MDD and GAD (22%). Even though co-occurance of CM and psychiatric disorders indicates a possible relationship, it is not necessarily causative [42]. Studies looking into this connection usually emphasize a shared biological mechanism between depression/anxiety and migraine, in which some common neurotransmitters (especially serotonin) operate [43].

Additionally problems caused by headaches which these patients experience during their daily lives (such as feelings of loss of control, disturbances in academical and social functioning) may contribute to the development of psychiatric problems. As previously mentioned, headache symptoms limit and negatively affect daily activities of these patients; thus result in psychiatric disorders and decreased quality of life. In the literature, most of the studies which used Pediatric Quality of Life Inventory (PedsQL) report that children and adolescents who are diagnosed with migraine have worse quality of life compared to healthy controls [44–46]. However, results of the studies which examined quality of life using PEDMIDAS are contradicting: There are studies which states; significant difference between migraine and health control groups, significant difference only for migraine patients with comorbid psychiatric disorders and no significant difference at all [41, 47]. In our study, we could not determine any clinical or psychiatric (except for attention problems and somatic symptoms) parameter which might be related to lower PEDMIDAS scores (Tables 2 and 5). Several reasons may contribute to the fact that studies which use PEDMIDAS (like ours) fail to replicate the common findings of the studies which use PedsQL in adolescents with migraine. It has been previously defined that understandability and practicality of PedMIDAS get lower as the age decreases [47]. Furthermore; even though items of of PEDMIDAS have universal characteristics and it has previously been used in studies conducted on Turkish patients, there is no reliability and validity study of PEDMIDAS in Turkish language [17]. PedMIDAS evaluates loss of functionality in an objective manner (according to the number of the days that the patient felt restricted) but overlooks subjective features (like decrease in the motivation/ capacity and feelings of distress) and this may also contribute to those contradicting findings [48]. In this respect, it can be stated that using PedsQL (which is based on more subjective definitions) instead of PEDMIDAS might be more suitable for the studies which examine functionality of children and adolescents.

**Table 5:** Correlations between DSM-5 Level 1 Cross-Cutting Symptom Measure (CCSM-5) scores and Visual Analogue Scale (VAS)/ Pediatric Migraine Disability Assessment Scale (PEDMIDAS) scores

CCSM-5 Symptom Cluster	VAS		PEDMIDAS	
	r	p^a^	r	p^a^
**Somatic Symptoms**	0.143	0.320	**0.399**	**0.004**
**Sleep Problems**	0.102	0.482	0.026	0.858
**Attention Problems**	**0.388**	**0.005**	**0.348**	**0.013**
**Depressive Symptoms**	0.152	0.292	0.112	0.438
**Irritability**	−0.157	0.278	−0.143	0.322
**Agression**	0.016	0.911	−0.099	0.493
**Manic Symptoms**	−0.017	0.904	0.107	0.459
**Anxiety Symptoms**	−0.081	0.575	0.018	0.903
**Psychotic Symptoms**	−0.023	0.875	0.188	0.191
**Repetitive Thoughts/ Behaviors**	−0.196	0.173	0.074	0.609

*r* Pearson Correlation Coefficient

^a^ Statistically significant values are written in bold

In their meta-analysis, Balottin et al. (2013) have found that adolescents with migraine have higher scores not only in internalizing symptoms (depression and anxiety) but also in externalizing symptoms and total problem scores which suggest that externalizing problems also play critical roles on migraine symptomatology [49]. Even though effects of externalizing disorders on migraine are throughly known, definitions on their relationship are rare [42, 50]. Main externalizing disorder which is previously linked to migraine is ADHD [39]. Study of Arruda et al. which is also one of the few studies on migraine conducted using DSM-5 criteria, showed that prevalence of ADHD among adolescents with CM is relatively higher [51]. This co-occurrence of ADHD and CM is generally explained with common physiopathological processes such as disturbances in common dopamine neurotransmitter pathways, decrease in brain iron levels and sleep problems [52]. In line with previously mentioned the meta-analysis of Balottin et al. (2013), we also found that adolescents with CM scored higher on externalizing symptoms (sleep problems, attention problems, aggression, irritability and manic symptoms) according to CCSM-5; but they did differ on thresholds of manic symptoms and attention problems clusters (Table 4. Kristjándóttir reported that children suffering from frequent pains have significantly higher anger levels [53]. In another study which Arruda and colleagues done in 2010; instead of migraine and ADHD comorbidity, they defined an association between migraine and hyperactive-impulsive behavior pattern which might also explain the higher aggression/ irritability scores in our sample [54]. Furthermore, studies which emphasize the role of sleep disturbances in the context of migraine-ADHD co-occurrence are parallel with our patients’ higher sleep problem scores on CCSM-5 [52]. In our research, adolescents with CM did not differ from healthy controls in regard of exceeding threshold for attention problems or not; but rates of individuals exceeding tresholds for sleep problems, aggression and irritability were significantly higher in the case group. Our finding supports the hypothesis which proposes that association between migraine and ADHD operates via hyperactive-impulse behavior pattern and sleep disturbances. Additionally; we found positive correlations between attention problem scores on CCSM-5 and VAS (r = 0.388, *p* = 0.005, Pearson correlation)/ PEDMIDAS (r = 0.348, *p* = 0.013, Pearson correlation) scores (Table 5). Our findings may indicate a negative effect of hypersensitized pain axis among individuals with higher functional loss and headache severity on cognitive functions. In fact, neuropsychological and neuroimaging studies in this aspect reported that attentional control effect of visual cortex is altered and orientation response towards acoustic stimuli is decreased among patients with migraine [55].

This study only included adolescents CM patients; thus it was conducted on a fairly homogenous clinical headache sample which can be counted as its methodological strength. Also it is a rather detailed clinical research in which numerous individual and environmental risk factors and their effects on functionality, headache severity and psychiatric symptomatology were examined by both a neurologist and a child and adolescent psychiatrist. Furthermore, studies on adolescent migraine patients which used DSM-5 criteria are limited; implementation of ICHD-3 beta version and DSM-5 criteria in order to examine the association between CM and psychopathology in adolescent age group is the major strength of our research. Despite its important findings and strenghts, our study also has some limitations. Firstly, we did not examine clinical and environmental risk factors for migraine in control group; so it was not the main of this study, it could not be determined if these risk factors really effect pathophysiology of migraine or not. Secondly, psychiatric screening tool that we used (CCSM-5) only evaluates the symptoms during last 2 weeks and instruments which examine psychosymptomatology in a longer time period may better explain the psychopathological processes underlying CM. Thirdly, cross-sectional design and relatively small sample size of our study might make it hard to fully clarify a cause and effect relationship between clinical conditions and generalize its results to a larger population. In addition we would like to add that, even though our sample size is sufficient according to the power analysis; further studies with larger sample sizes are needed in order to portray a more defined framework about the relationship between CM and psychological well-being.

In conclusion, migraine is a debilitating neurological condition which disturbs daily activites, quality of life and psychological well-being of adolescents. In order to fully understand the mechanisms leading to this rather enigmatic association between migraine and psychiatric problems, futher studies with larger sample size and longitudinal design which use DSM-5 criteria are needed. We believe that revealing this relationship will enhance a multidisciplinary treatment approach which will cover somatic, cognitive and psychiatric aspects of the clinical complaints in adolescents with CM. Thereby, management of these conditions and pain will become easier and rational medication modalities will have positive effects on both individual and population-wide economies. Another point which should be emphasized is the need of detailed anamnesis for CM patients who were referred to emergency departments. Acquiring comprehensive psychological and neurological background of CM patients in emergency departments will not only guide effective treatment modalities; but also prevent negative radiation exposure and economical effects of unnecessary imaging techniques like x-rays or CTs.

### Ethical statement of research

The study was conducted in accordance with the ethical guidelines, including the World Medical Association Declaration of Helsinki 2008, and the legal requirements of the Ethics Committee of the institution it was conducted in (approval no: 2020/99). All methods were carried out in accordance with relevant guidelines and regulations. Informed consent was obtained from from a parent. All experimental protocols were approved by a named institutional committee.

## Data Availability

The datasets used and/or analysed during the current study available from the corresponding author on reasonable request.

## References

[1] Genizi J, Srugo I, Kerem NC (2013). The cross- ethnic variations in the prevalence of headache and other somatic complaints among adolescents in northern Israel. J Headache Pain.

[2] King S (2011). The epidemiology of chronic pain in children and adolescents revisited: a systematic review. Pain.

[3] Hammond NG, Orr SL, Colman I (2019). Early life stress in adolescent migraine and the Mediational influence of symptoms of depression and anxiety in a Canadian cohort. Headache.

[4] Lipton RB (2011). Prevalence and burden of chronic migraine in adolescents: results of the chronic daily headache in adolescents study (C-dAS). Headache.

[5] Abu-Arafeh I (2010). Prevalence of headache and migraine in children and adolescents: a systematic review of population-based studies. Dev Med Child Neurol.

[6] Headache Classification Committee of the International Headache Society (IHS) The International Classification of Headache Disorders, 3rd edition. Cephalalgia. 2018;38(1):1–211. 10.1177/0333102417738202. 10.1177/0333102417738202 29368949

[7] Bigal ME (2004). Primary chronic daily headache and its subtypes in adolescents and adults. Neurology.

[8] Blume HK (2017). Childhood headache: a brief review. Pediatr Ann.

[9] Wiech K, Tracey I (2009). The influence of negative emotions on pain: behavioral effects and neural mechanisms. Neuroimage.

[10] Langdon R, DiSabella MT (2017). Pediatric headache: an overview. Curr Probl Pediatr Adolesc Health Care.

[11] Pawlowski C (2019). National Trends in pediatric headache and associated functional limitations. Clin Pediatr (Phila).

[12] Stevens J, Hayes J, Pakalnis A (2014). A randomized trial of telephone-based motivational interviewing for adolescent chronic headache with medication overuse. Cephalalgia.

[13] Charles JA (2010). Chronic daily headache in adolescents: an 8-year follow-up study. Neurology.

[14] Wang SJ (2006). Chronic daily headache in adolescents: prevalence, impact, and medication overuse. Neurology.

[15] Carasco M, Kroner-Herwig B (2016). Psychological predictors of headache remission in children and adolescents. Adolesc Health Med Ther.

[16] Galli F (2017). Headache and psychological disorders in children and adolescents: a cross-generational study. Minerva Pediatr.

[17] Kandemir G, Hesapcioglu ST, Kurt ANC (2018). What are the psychosocial factors associated with migraine in the child? Comorbid psychiatric disorders, family functioning, parenting style, or Mom's psychiatric symptoms?. J Child Neurol.

[18] Tarantino S (2013). Clinical features, anger management and anxiety: a possible correlation in migraine children. J Headache Pain.

[19] Faul F (2007). G*power 3: a flexible statistical power analysis program for the social, behavioral, and biomedical sciences. Behav Res Methods.

[20] Ozturk A (2008). Reference body mass index curves for Turkish children 6 to 18 years of age. J Pediatr Endocrinol Metab.

[21] Lucas C (2012). Stability, responsiveness, and reproducibility of a visual analog scale for treatment satisfaction in migraine. Headache.

[22] Topcu Y (2014). The Paediatric migraine disability assessment score is a useful tool for evaluating prophylactic migraine treatment. Acta Paediatr.

[23] Heyer GL (2014). PedMIDAS-based scoring underestimates migraine disability on non-school days. Headache.

[24] Vahia VN (2013). Diagnostic and statistical manual of mental disorders 5: a quick glance. Indian J Psychiatry.

[25] Sapmaz SY (2018). Validity and reliability of the Turkish version of DSM-5 level 2 anxiety scale (child form for 11-17 years and parent form for 6-17 years). Noro Psikiyatr Ars.

[26] Kaufman J (1997). Schedule for affective disorders and schizophrenia for school-age children-present and lifetime version (K-SADS-PL): initial reliability and validity data. J Am Acad Child Adolesc Psychiatry.

[27] Caye A (2017). Schedule for affective disorders and schizophrenia for school-age children - present and lifetime version (K-SADS-PL), DSM-5 update: translation into Brazilian Portuguese. Braz J Psychiatry.

[28] Unal F (2019). Reliability and Validity of the Schedule for Affective Disorders and Schizophrenia for School-Age Children-Present and Lifetime Version, DSM-5 November 2016-Turkish Adaptation (K-SADS-PL-DSM-5-T). Turk Psikiyatri Derg.

[29] Caperell K, Pitetti R (2014). Seasonal variation of presentation for headache in a pediatric emergency department. Pediatr Emerg Care.

[30] Torres-Ferrus M (2019). Headache, comorbidities and lifestyle in an adolescent population (the TEENs study). Cephalalgia.

[31] Wiendels NJ (2005). Chronic daily headache in children and adolescents. Headache.

[32] Goubert L (2011). Learning about pain from others: an observational learning account. J Pain.

[33] Casey K. Sensory, Motivational, and Central Control Determinants of Pain. 1968:423–39. 10.1590/1516-4446-2017-2317.

[34] Merikangas KR, Angst J, Isler H (1990). Migraine and psychopathology. Results of the Zurich cohort study of young adults. Arch Gen Psychiatry.

[35] Blaauw BA (2015). The relationship of anxiety, depression and behavioral problems with recurrent headache in late adolescence - a young-HUNT follow-up study. J Headache Pain.

[36] Kanner AD (1981). Comparison of two modes of stress measurement: daily hassles and uplifts versus major life events. J Behav Med.

[37] Pompili M (2009). Psychiatric comorbidity in patients with chronic daily headache and migraine: a selective overview including personality traits and suicide risk. J Headache Pain.

[38] Cahill CM, Cannon M (2005). The longitudinal relationship between comorbid migraine and psychiatric disorder. Cephalalgia.

[39] Arruda M (2017). Comorbidity with Psychiatric Disorders.

[40] Arita JH (2013). Adolescents with chronic migraine commonly exhibit depressive symptoms. Acta Neurol Belg.

[41] Oztop DB (2016). Assessment of psychopathology and quality of life in children and adolescents with migraine. J Child Neurol.

[42] Guidetti V, Galli F, Sheftell F (2010). Headache attributed to psychiatric disorders. Handb Clin Neurol.

[43] Ferrari MD, Saxena PR (1993). On serotonin and migraine: a clinical and pharmacological review. Cephalalgia.

[44] Brna PM, Dooley JM (2006). Headaches in the pediatric population. Semin Pediatr Neurol.

[45] Powers SW (2004). Quality of life in paediatric migraine: characterization of age-related effects using PedsQL 4.0. Cephalalgia.

[46] Talarska D, Zgorzalewicz-Stachowiak M (2007). The influence of selected factors on the quality of life of children with headaches. Adv Med Sci.

[47] Hershey AD (2001). PedMIDAS: development of a questionnaire to assess disability of migraines in children. Neurology.

[48] Kroner-Herwig B, Heinrich M, Vath N (2010). The assessment of disability in children and adolescents with headache: adopting PedMIDAS in an epidemiological study. Eur J Pain.

[49] Balottin U (2013). Psychopathological symptoms in child and adolescent migraine and tension-type headache: a meta-analysis. Cephalalgia.

[50] Virtanen R (2004). Externalizing problem behaviors and headache: a follow-up study of adolescent Finnish twins. Pediatrics.

[51] Arruda MA (2020). ADHD is comorbid to migraine in childhood: a population-based study. J Atten Disord.

[52] Paolino MC (2015). Headache and ADHD in pediatric age: possible Physiopathological links. Curr Pain Headache Rep.

[53] KristjÁNsdÓTtir G (1997). The relationship between pains and various discomforts in SchoolChildren. Childhood.

[54] Arruda MA (2010). Migraine, tension-type headache, and attention-deficit/hyperactivity disorder in childhood: a population-based study. Postgrad Med.

[55] Leveque Y, et al. Self-perceived attention difficulties are associated with sensory hypersensitivity in migraine. Rev Neurol (Paris). 2020. 10.1016/j.neurol.2020.01.360. 10.1016/j.neurol.2020.01.360 32312498

